# Phylogenetic diversity and functional potential of large and cell-associated viruses in the Bay of Bengal

**DOI:** 10.1128/msphere.00407-23

**Published:** 2023-10-30

**Authors:** Benjamin Minch, Salma Akter, Alaina Weinheimer, M. Shaminur Rahman, Md Anowar Khasru Parvez, Sabita Rezwana Rahman, Md Firoz Ahmed, Mohammad Moniruzzaman

**Affiliations:** 1 Department of Marine Biology and Ecology, Rosenstiel School of Marine, Atmospheric, and Earth Science, University of Miami, Miami, Florida, USA; 2 Department of Microbiology, Jahangirnagar University, Dhaka, Bangladesh; 3 Bigelow Laboratory for Ocean Science, East Boothbay, Maine, USA; 4 Department of Microbiology, Jashore University of Science and Technology, Jashore, Bangladesh; 5 Department of Microbiology, University of Dhaka, Dhaka, Bangladesh; University of Michigan, Ann Arbor, Michigan, USA

**Keywords:** marine viruses, giant virus, jumbo phages, aquatic virome, viral ecology, Bay of Bengal, marine microbiology, Indian Ocean, Bay of Bengal virome

## Abstract

The Bay of Bengal (BoB) is the world’s largest bay, offering essential services like fishing and recreation while holding significant economic value for coastal communities. However, the BoB faces environmental challenges from monsoons, freshwater inputs, rising sea levels, and intensified cyclones due to climate change. Human activities such as tourism and development also impact the region, necessitating a global change perspective. Despite its importance, microbial diversity and ecology in the BoB remain largely unexplored. We focused on large and cell-associated viruses (i.e., originating from the cellular size fraction), particularly giant viruses and large phages in two BoB coastal sites: Cox’s Bazar, a populated beach with freshwater influences, and Saint Martin Island, a less affected resort island. Metagenomic sequencing reveals a higher abundance and diversity of viruses in Cox’s Bazar and presence of viruses that suggest freshwater intrusion and runoff. We identified 1962 putative phage genomes (10–655 kbp). Notably, 16 “large” phages >100 kbp were found in Saint Martin, and a terminase large subunit marker gene phylogeny revealed substantial diversity of large phages along the BoB coast. The BoB virome encodes diverse functionalities, with a greater presence of auxiliary metabolic genes in the Cox’s Bazar viral community. Additionally, five giant virus genomes (phylum Nucleocytoviricota) encoding various functionalities are reconstructed from Cox’s Bazar (83–876 kbp). This pioneering study revealing the viral diversity and host interactions in coastal BoB lays the foundation for future investigations into viral impact on biogeochemical cycles and the microbial food web in this understudied environment.

**IMPORTANCE:**

The BoB, the world’s largest bay, is of significant economic importance to surrounding countries, particularly Bangladesh, which heavily relies on its coastal resources. Concurrently, the BoB holds substantial ecological relevance due to the region’s high vulnerability to climate change-induced impacts. Yet, our understanding of the BoB’s microbiome in relation to marine food web and biogeochemical cycling remains limited. Particularly, there are little or no data on the viral diversity and host association in the BoB. We examined the viral community in two distinct BoB coastal regions to reveal a multitude of viral species interacting with a wide range of microbial hosts, some of which play key roles in coastal biogeochemical cycling or potential pathogens. Furthermore, we demonstrate that the BoB coast harbors a diverse community of large and giant viruses, underscoring the importance of investigating understudied environments to discover novel viral lineages with complex metabolic capacities.

## INTRODUCTION

The Bay of Bengal (BoB) is the largest bay in the world and provides ecological and economic services to all surrounding nations. Home to many unique habitats such as seagrass beds, coral reefs, and mangrove forests, the BoB is an important biodiversity hotspot (1). The diversity of habitats and species in BoB has made it an important ecotourism destination in surrounding nations, with many countries developing their tourism economies around activities in the bay (2, 3). In addition to ecotourism, the BoB is also a source of many natural resources. One such resource is the many fisheries along the coast of the BoB (4). These fisheries have been described as some of the most productive in the world, given the wide range of species and the vast area of water in the bay (5). In Bangladesh, these fisheries are the second largest source of employment, making up 8% of the workforce and employing around 13 million people (6). These fisheries are supported by a large population of coastal mangrove habitats as well as a diverse assemblage of microbes making up the bottom of the food web (7, 8).

Despite its importance, the Bay of Bengal is one of the most understudied bodies of water in the world (9). Consequently, the microbial communities at the foundation of the BoB ecosystem remain poorly understood. A few recent studies have looked at bacterial diversity through 16S amplicon sequencing, but very few have looked at the functional potential and interactions of the microbial communities in this region (10, 11). Among the studies that have attempted to look at microbial diversity through metagenomics, all have been focused on sediment bacteria in the deep sea (12), with little or no efforts targeted toward understanding the microbiota of surface water communities, the communities closely associated with corals, seagrass, and mangroves using similar approach. Unlike taxonomic-focused studies that exclusively enumerate which species are present, metagenomic studies provide functional information about these microbial communities related exclusively to their role in the broader biogeochemical cycling of the ecosystems. It is important to note, however, that functional information derived from metagenomics alone can only make functional predictions, as further metatranscriptomic and proteomic approaches would be needed to confirm transcription and translation of a functional protein.

Another substantial gap in our understanding of the microbial dynamics of BoB is that all previous studies on the microbial community have excluded viruses. The one exception to this is the study of the white spot syndrome virus, a virus infecting black tiger shrimp, important aquaculture species in the BoB (13). Viruses are the most abundant biological entities in the ocean and influence nutrient cycling, biogeochemical cycles, population dynamics of prokaryotes and microeukaryotes, algal bloom dynamics, and numerous other ecological processes (14). It is estimated that there are about 10^9^ viruses per liter of water and that they are responsible for the death of roughly 10%–50% of the bacteria in the surface ocean every day (14, 15). In addition to simply killing their host, many viruses have been shown to modulate host metabolism through the use of auxiliary metabolic genes (AMGs) (16). These AMGs have large implications for molecular biogeochemical cycling as they have the potential to alter host nutrient uptake, photosynthesis, and carbon metabolism (17).

While most viruses likely contain AMGs, certain viruses have much larger genomes and potentially harbor more metabolic capacities to modulate host metabolism. Jumbo phages, bacteriophages with large genomes and large capsids, are prime examples of this as their large genomes can potentially contain more AMGs than an average smaller bacteriophage that has fewer genes (18). These jumbo phages have until recently been underrepresented in virome data due to their large size (upwards of 0.45 μm) and filtering strategies commonly adopted in environmental virome studies which typically sequence filtrate passing through 0.22-μm filters (19–21). Another group of large viruses is the nucleocytoplasmic large DNA viruses (NCLDVs). These DNA viruses that comprise the phylum Nucleocytoviricota mainly infect microbial eukaryotes (also known as protists), typically have large genomes (upwards of 2.5 Mb), have large virion sizes (upwards of 1.5 μm) (22), and are widespread in the Earth’s oceans (23, 24). NCLDVs have received recent attention due to their wide genomic repertoire and potential for metabolic reconstruction in their host (24, 25). Both jumbo phages and NCLDVs present a huge potential for housing a large number of AMGs and thus have a substantial effect on host metabolism and biogeochemical cycles.

Viruses, along with other members of the microbial community, have been shown to shift their dynamics and diversity in response to various environmental and anthropogenic stressors (26, 27). These stressors are abundant in the BoB as many of the bordering countries are developing at fast rates and still lack basic environmental regulations present in more developed nations. For example, the regulation of wastewater remains a large problem for coastal communities along the bay. With large populations and underdeveloped wastewater treatment plants, much of the household and industrial wastewater makes its way into rivers and the bay (28). Additionally, countries surrounding the BoB are hit by frequent monsoons which lead to agricultural runoff of fertilizers and pesticides (29). This runoff, along with other environmental stressors, has the potential to shift the microbial community as nutrients and waste products are added to the waterways (30, 31).

Furthermore, understanding the effects of environmental stressors on microbial communities in the BoB requires an examination of the viral diversity and its association with host organisms. Despite the potential impact of runoff water on microbial population dynamics, the viral community in this region and its interactions with hosts have yet to be characterized. Addressing this gap, our study reports the composition and dynamics of the viral community at two distinct sites on the eastern side of the Bay of Bengal, Bangladesh. Specifically, using metagenomic data, we sought to identify the large and cell-associated viruses (viruses present in the cellular size fraction) present at these sites to characterize the diversity and abundance of viral populations in this understudied ecosystem. Functional annotations and host predictions were also performed to examine the roles these viruses could be playing in the metabolism of the respective microbial communities. To our knowledge, this is the first characterization of the cell-associated viral populations in this region. This foundational work is crucial to inform future investigation and ecological characterization of this unique ecosystem and its interplay with the developing human civilizations around it.

## MATERIALS AND METHODS

### Sample collection, processing, and sequencing

**Fig 1:**
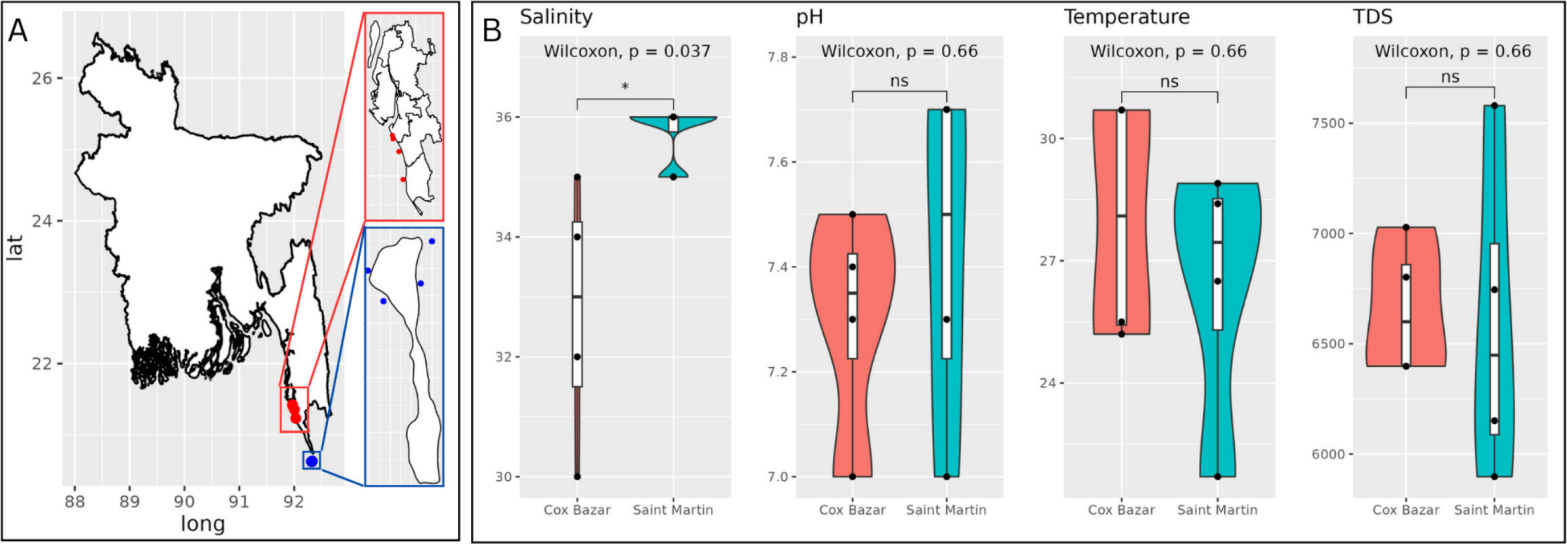
Sampling location and physicochemical parameters. (**A**) A map of Bangladesh and study locations. Cox’s Bazar is highlighted in red, and Saint Martin is highlighted in blue. Map was constructed in R using ‘ggmap’ package. (**B**) Physicochemical measurements of the two sites. Violin plots represent the measurements from four locations at each site with a boxplot in the center showing the mean and range of the data. Sites were compared using Wilcoxon test. * indicates statistically significant difference (*P*-value < 0.05). ns, not significant; TDS, total dissolved solid.

Samples were collected from four different sites at both Saint Martin and Cox’s Bazar, Bangladesh. These four sites were aggregated into a single sample for each location which from this point forward will be referred to as simply Saint Martin and Cox’s Bazar. These were collected from seawater between 2 and 3 March 2022 during low tide. Samples were collected in 1-L sterile sampling bottles at a 1.5-m depth from the surface. After collection, samples were processed at the microbiology laboratory at Jahangirnagar University, Savar, Dhaka, Bangladesh. The geographic location of sampling, as well as the measured environmental parameters, are shown in Fig. 1. A Wilcoxon test was performed to test for significant differences in these environmental parameters.

Water samples were first passed through Whatman filter paper no. 1 (pore size 11 μm) and then subsequently passed through a 0.45-μm membrane and 0.2-μm membrane. From each of these two filters, DNA was extracted using the DNeasy PowerWater Kit (QIAGEN) according to the manufacturer’s protocol. Purified DNA samples from each membrane were mixed together and sent to EzBiome Inc., USA, for metagenomic sequencing. An equal quantity of DNA from both membranes from the four representative sites were pooled together as a single sample from each site.

Paired-end (2 × 150 bp) sequencing was performed using a Novaseq 6000 sequencer (Illumina Inc., USA). The FASTQ files were evaluated for quality using FASTQC (v.0.11) (32) and adapter sequences and low-quality ends were trimmed using Trimmomatic (v.0.39) (33). After trimming, the read counts for Saint Martin and Cox’s Bazar were 33.94 and 31.8 million, respectively, corresponding to 92.2% and 92.37% of total reads. Negative controls were used in both sequencing and DNA extraction. Sequencing and assembly statistics are shown in Table S1.

### Identification and characterization of the phage community

Metagenomic reads from both sites were trimmed using Cutadapt (v.4.4) (34) and then assembled using MetaSpades (v.3.15.5) (35). These assemblies from Saint Martin and Cox’s Bazar were then analyzed using the ViWrap pipeline (v.1.2.1) with default settings to bin viral contigs as well as classify them and predict their functional capacities (36). ViWrap uses a combination of Virsorter2 (v.2.4.5), VIBRANT (v.1.2.1), and DeepVirFinder (v.2020.11.21) to identify viral contigs. Contigs are then binned using vRhyme (v.1.1.0). Phages are then classified using the VOG HMM database (VOG 97) and National Center for Biotechnology Information (NCBI) RefSeq (release 218) viral protein database. Host prediction was also performed in this pipeline using the iPHOP module (v.1.2.0).

Viral contigs obtained from ViWrap were de-replicated using dREP (v.3.4.0) (37) with a 95% average nucleotide identity (ANI) cutoff and quality assessed with CheckV, resulting in a total of 1962 viral sequences (a mix of binned and unbinned contigs) that had a CheckV quality assignment of medium or higher. This set of de-replicated sequences was used for read mapping from both sites. Read mapping was done using minimap2 (v.2.24) (38), and then coverage was determined using CoverM (in ‘genome’ mode) with a minimum identity of 95% (v.0.6.1) (39). Graphs of viral abundance using [reads per kilobase per million (RPKM)] were generated using ggplot2 in R. Shannon diversity was also calculated using the vegan R package, treating de-replicated viral bins and unbinned contigs as “viral populations.” This index was bootstrapped 1,000 times, and a Welch *t*-test was used to determine significance.

### Phylogenetic analysis

For major capsid protein (mcp) phylogenetic analysis of NCLDV, mcp proteins belonging to NCLDV members were identified using NCLDV markersearch script (24). This set of mcp proteins was then de-replicated at 95% using cd-hit (v.4.8.1) (40). Sequences from mcp were aligned to a reference set of MCP proteins from known NCLDV derived from the Giant Virus Database (41). MAFFT was used for alignment (v.7.511) (42) using default parameters. Aligned sequences were then made into a maximum likelihood tree using fasttree with default settings (v.2.1) (43). The tree was visualized using iTol (44).

Phylogeny of the terminase large subunit (*terL*) gene found in bacteriophages was reconstructed using sequences derived from our data as well as multiple different databases. Reference sequences were obtained from the VOG database (http://vogdb.org/), NCBI Refseq database (45), the infrared database (46), and data from Al-Shayeb et al. (19) and Weinheimer et al. (20). Metadata on genome size, location of isolation, and identification can be found in the supplemental information (see Data Availability statement). *terL* sequences from our data set were identified using HMMER3 (v.3.3.2) (47) with an *E*-value cutoff of 1e-5 against all the *terL* HMM profiles from the VOG database. All of these sequences were clustered at 95% ANI using cd-hit and aligned with MAFFT using the auto setting. The alignment was then trimmed using TrimAL (v.1.3) (48) with parameter “-gt 0.1,” and a phylogenetic tree was reconstructed using IQ-Tree (v.2.2) (49) with an LG + F + R10 model. The tree was visualized using iTol (44). The alignment length was 1,463, and the tree was bootstrapped 100 times. A tree with the bootstrap values is available in the supplemental data provided.

### NCLDV genome binning and contig prediction

A standardized pipeline was used to identify and bin NCLDV genomes in our data set. For NCLDV functional analysis, assembled contigs were binned using metabat2 (v.2.12.1) (50). Proteins were predicted for the bins using prodigal-gv (v.2.11) (51), and then bins were screened for the presence of NCLDV marker genes using the NCLDV markersearch script. Bins with at least one hit to an NCLDV marker gene (*mcp, sfII, rnapS, rnapL, polB, tfIIB, topoII, a32*, and *vltf3*) were kept and screened for signatures of NCLDV contigs using ViralRecall (41). Bins with a positive ViralRecall score were kept for further screening which involved the removal of bins not fitting the criteria of having three of the four key marker genes described by Aylward et al. (52). After screening, contigs with negative ViralRecall scores were removed from the bins as they most likely represent bacterial or eukaryote contamination. Following this stringent protocol resulted in five NCLDV genomes. tRNAs for these genomes were predicted with tRNAscan-SE (53).

### Functional analysis

Functional annotations of the genes in the NCLDV genomes were assigned using HMMER3 against the GVOG (52), Pfam (54), and EGGNOG (55) databases using an *E*-value cutoff of 1e−5. Viral contigs that were not binned into a genome were also identified with ViralRecall and annotated in a similar fashion with HMMER3. In total, there were 60 contigs found in Cox’s Bazar and 39 found in Saint Martin that were not present within the NCLDV metagenome-assembled genome (MAGs). Functional categories were then graphed as a proportion of total functionality using ggplot2. Maps of giant virus genomes were also made with the circlize(v.0.4.15) (56) package in R.

For prediction of gene functionalities of phages, the ViWrap pipeline was used, which employs VIBRANT to categorize AMGs of viral contigs. After running ViWrap for each site, AMGs coming from large phages (those with genomes or unbinned contig sizes over 100 kbp) were separated out for comparison. Resulting functional plots were made using the ggplot2 package in R.

## RESULTS

### Diversity, abundance, and functional potential of prokaryotic viruses

We obtained a total of 1962 phage viral genomes from both Saint Martin’s Island and Cox’s Bazar that passed the screening criteria of ViWrap (see Materials and Methods). Of these, 1,058 (53.92%) were unique to Cox’s Bazar and 843 (42.97%) were unique to Saint Martin at a 95% ANI cutoff. Sixty-one genomes were shared between sites (3.01%).

**Fig 2:**
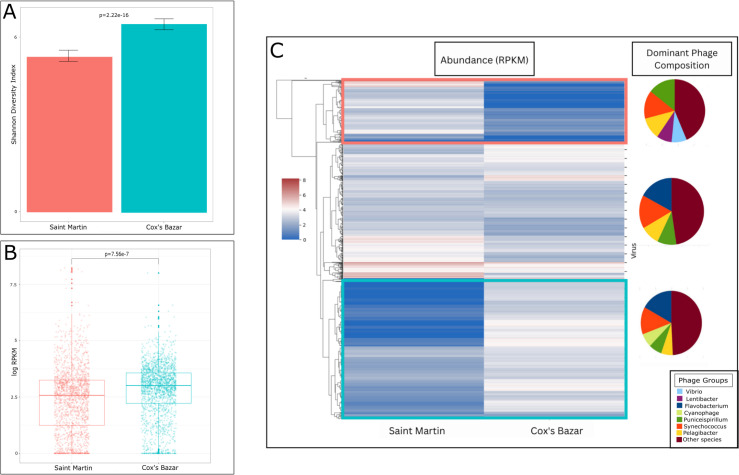
Prokaryotic viral abundance and diversity. (**A**) Shannon diversity indices were calculated from viral populations at both sites. Error bars represent ±2 standard errors of the mean. (**B**) Comparison of the overall abundance of prokaryotic viruses at each site. Each dot represents a virus and its measured abundance (reads per kilobase per million [RPKM]). A *t*-test was performed on the mean RPKM between sites (*P* = 7.56e-7). (**C**) Heatmap showing viral abundance and dominant phage composition of each cluster. Each line in the heatmap represents one virus and its abundance between the two sites. Three clusters were formed from this heatmap: viruses present almost exclusively in Saint Martin (red box), viruses present almost exclusively in Cox’s Bazar (blue box), and viruses present in both sites (no box). The dominant phage groups are shown for each of these three clusters, represented as a proportion of the total cluster population.

A comparison of alpha diversity between Saint Martin and Cox’s Bazar revealed Cox’s Bazar to have both a higher Shannon diversity (*P* = 2.2e-16) and relative abundance of viruses with mean log RPKM of 3.0 compared to 2.6 for Saint Martin (Fig. 2A and B). Many viruses exhibited site-specific abundance patterns and were categorized into three groups: those almost exclusively found in Saint Martin, those almost exclusively found in Cox’s Bazar, and those found at both sites. The dominant viruses at Saint Martin showed homology to phages that belong to species that infect *Puniceispirillum*, *Synechococcus*, *Pelagibacter*, *Vibrio,* and *Lentibacter*. Meanwhile, the phages unique to Cox’s Bazar were mainly composed of phages with homology to species that infect *Puniceispillium*, *Synechococcus*, *Pelagibacter*, *Flavobacterium*, and *Cyanobacteria* (Fig. 2C).

**Fig 3:**
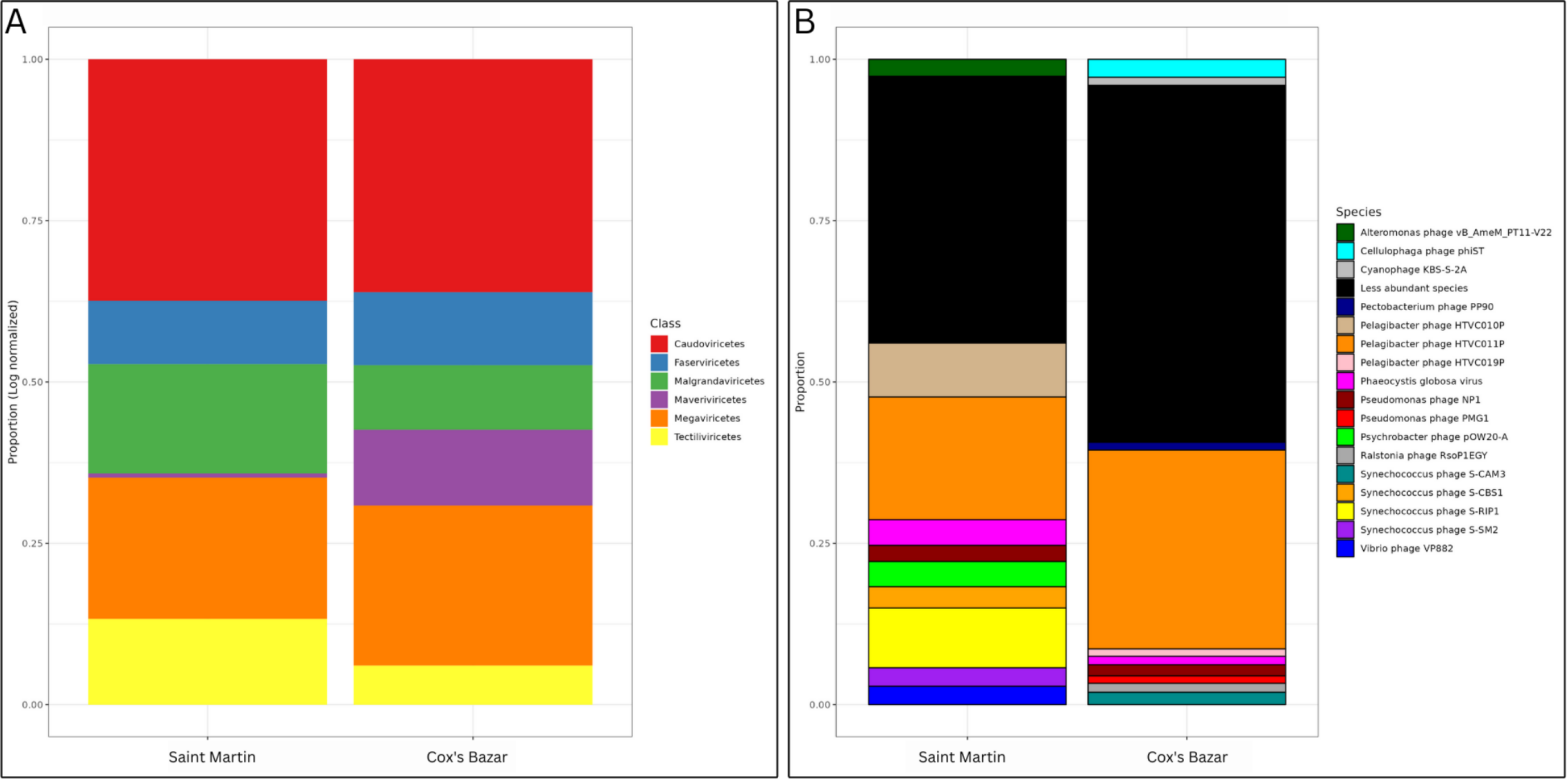
Viral community composition. (**A**) Class-level and (**B**) species-level taxonomic distributions of viruses between sites. Proportions are log normalized for class-level taxonomy to see better resolution. For the species-level taxonomy, only the top 10 most abundant species from each site are shown as a unique bar on the graph. All other species are grouped into the black bar titled “less abundant species.” Taxonomic assignments were obtained through the ViWrap pipeline.

Based on the DNA evidence recovered by our methods, in both sites, most of the DNA viruses were prokaryotic, belonging to the prokaryotic virus class of Caudoviricetes, followed by the giant virus class Megaviricetes. In general, the abundance of different viral classes was similar between sites with the exception of the virophage class Maveriviricetes as more prevalent in Cox’s Bazar and the Tectiliviricetes class, which includes prokaryotic viruses and adenoviruses of vertebrates, as more prevalent in Saint Martin (Fig. 3A).

When looking at the top 10 most abundant species (that were taxonomically identifiable) of phages in each site, there are some clear differences. Most of the phages in Saint Martin belonged to these top species, while the community of Cox’s Bazar was dominated by species that were rarer. The dominant viral species in both communities, however, shows homology to *Pelagibacter* phage HTVCO11P. Interestingly, Saint Martin hosts a larger proportion of phages showing homology to the species *Psychrobacter* phage pOW20-A than Cox’s Bazar, a phage normally found in cold or Antarctic waters. Saint Martin also has a larger proportion of phages with homology to species of *Vibrio* and *Alteromonas* phages than Cox’s Bazar (Fig. 3B). Among other notable species found in high abundance at Cox’s Bazar were viruses showing homology to *Cellulophaga* phage phiST, Cyanophage, and *Ralstonia* phage.

**Fig 4:**
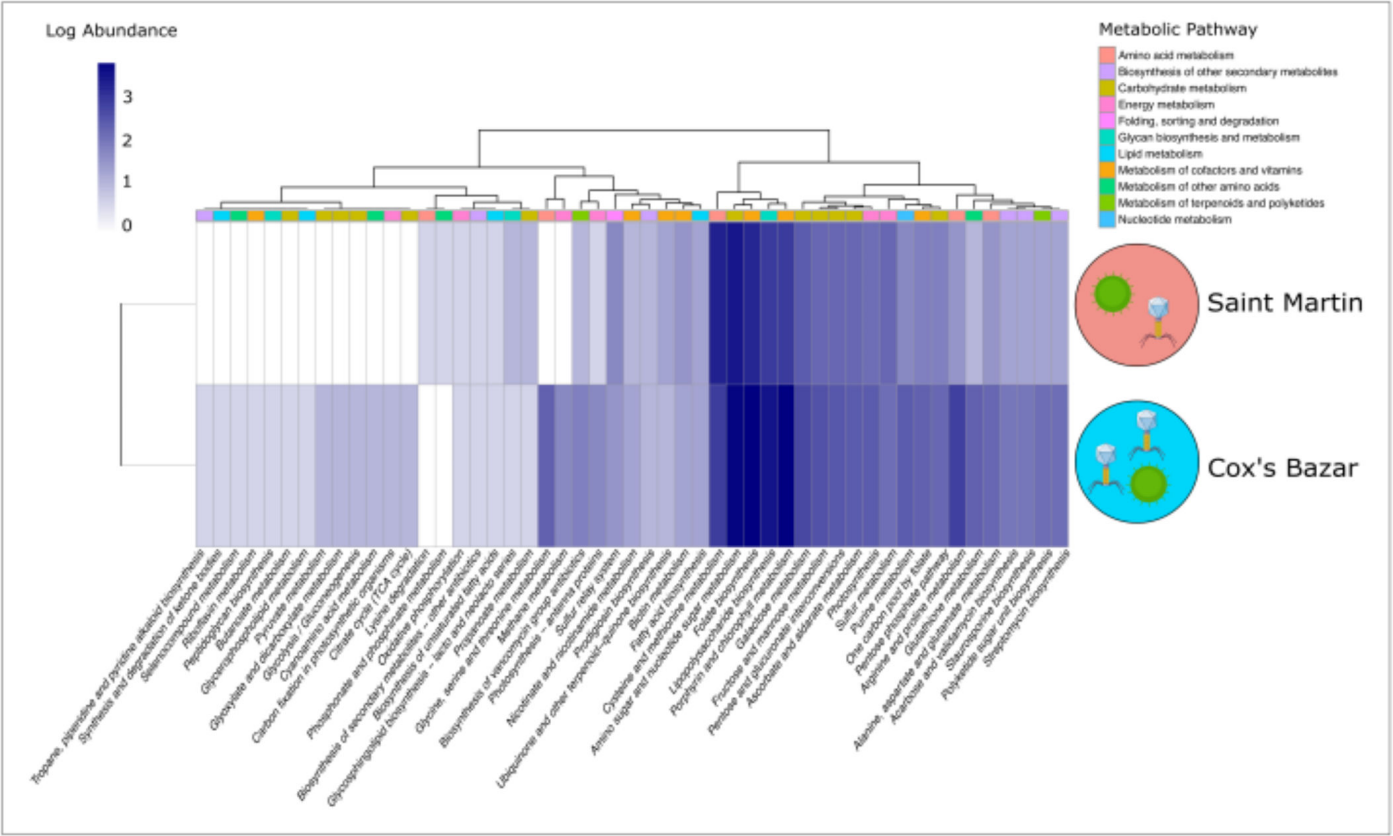
Functional profiling of prokaryotic viruses. A heatmap showing VIBRANT-predicted auxiliary metabolic genes (AMGs) of prokaryotic viruses from both locations. Values shown are log abundance of AMGs in each metabolic pathway, with larger metabolic categories being shown in the color strip at the top of the graph. Large phages are shown separately in Fig. S1.

Overall, 250 phage-encoded AMGs were identified in phages from Saint Martin, and 524 AMGs were identified from Cox’s Bazar (Fig. 4). Of these, 100 belonged to the genomes of large phages (>100 kbp). Most of these AMGs were categorized as being involved in the metabolism of cofactors and vitamins, with the most abundant pathways being the biosynthesis of folate and the metabolism of porphyrin and chlorophyll. A large number of AMGs putatively involved in cysteine and methionine metabolism, lipopolysaccharide biosynthesis, and amino sugar and nucleotide sugar metabolism were also found in these genomes. Cox’s Bazar had 15 more pathways that were not detected in Saint Martin’s Island, which included pathways involved in methane metabolism, glycolysis, the tricarbocylic acid (TCA) cycle, carbon fixation in photosynthetic organisms, pyruvate metabolism, glycine, serine, and threonine metabolism. Phosphonate metabolism and glycosphingolipid biosynthesis were the only pathways found to be unique to samples from Saint Martin.

### Large phage diversity and functional potential

The nomenclature for large phages is not yet definitive, likely because the size cutoffs used to define these groups have no evolutionary basis and these phages are likely paraphyletic, having evolved from smaller phages in multiple, independent occasions. For instance, phages with genomes over 200 kilobases have traditionally been called “jumbo phages,” but a recent study enumerating jumbo phages in diverse metagenomes referred to them as “huge phages” and created the term “megaphage” to refer to those with genomes over 500 kb (57). Although we recognize that most papers focused on jumbo phages refer to the 200-kbp cutoff (18, 20), for this study, we have opted to include all phages over 100 kbp in size in order to capture a broader range of these phages. For this work, we were primarily interested in identifying jumbo phages based on their phylogenies, and this cutoff was chosen to evaluate functional differences between large and smaller phages. Using this criterion, we identified 16 phages with genome sizes larger than 100 kbp from Cox’s Bazar, ranging from 102 to 655 kbp.

**Fig 5:**
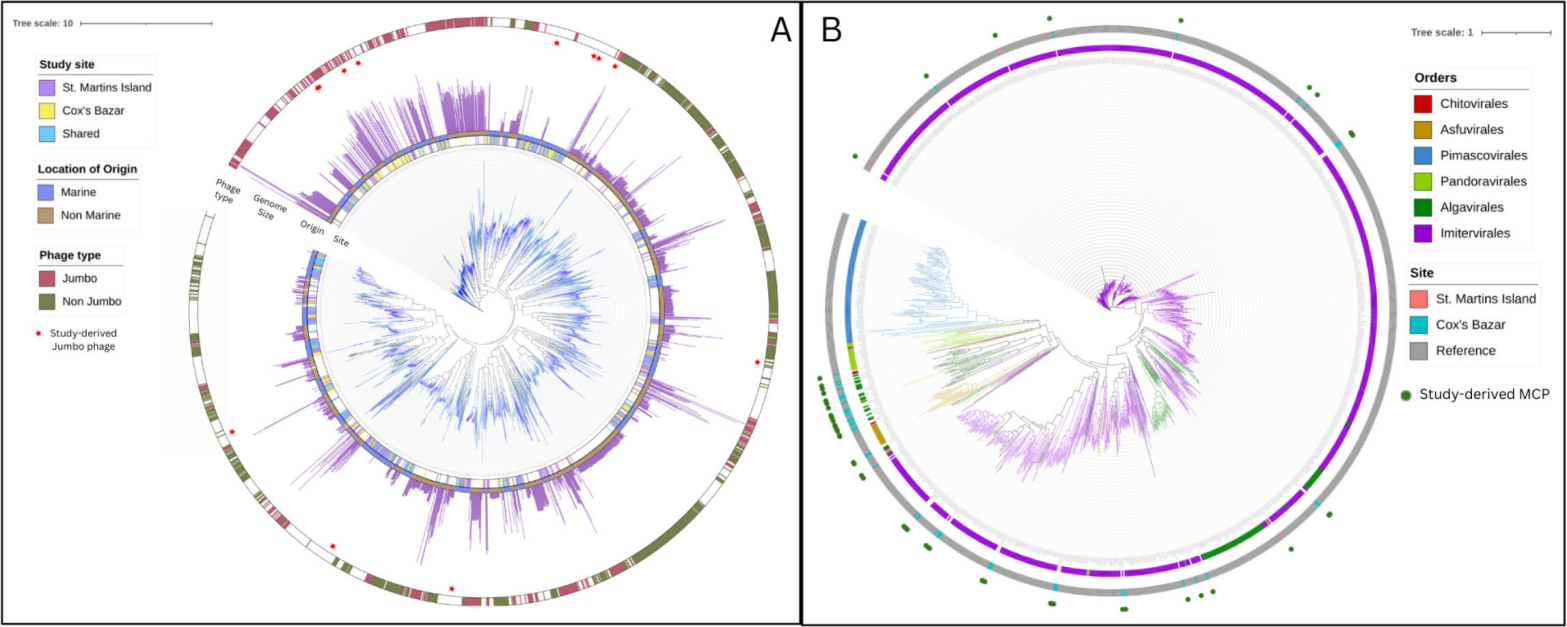
Phylogenetic analysis of large viruses. (**A**) Large phage phylogeny was constructed using terminase large subunit (*terL*) as a marker gene with the LG + F + R10 model in IQ-TREE. The tree includes all phages with a *terL* gene from our study as well as reference sequences from the VOG, NCBI Refseq, and the infrared database, as well as known jumbo phages from Al-Shayeb et al. (19), and Weinheimer and Aylward (20). (**B**) Phylogeny of the NCLDVs found in this study using the major capsid protein *(mcp*) as a phylogenetic marker. The maximum likelihood tree includes reference sequences from the Giant Virus Database. Strips showing the site where the *mcp* was obtained as well as order-level classification of reference sequences are present.

From our phylogenetic analysis of the *terL* gene, there emerged clades enriched with known large phages that cluster closely on the tree (Fig. 5A). Many of our own phage *terL* genes from Cox’s Bazar and Saint Martin also fell inside these clades, confirming the presence of jumbo phages in our sites and adding some support to the chosen 100-kbp cutoff. The remaining jumbo phages in our data set cluster close to other known jumbo phages on the tree, although not clustering inside of the enriched clades. There was generally no trend when it comes to site-specific clustering or isolation origin, although the jumbo clade is enriched in marine viruses with many non-marine jumbos clustering elsewhere.

Large phages had similar functional capacities to their smaller counterparts, having AMGs present in a broad range of categories despite there only being 16 identified genomes (Fig. S1). The only unique pathway found in these large viruses was thiamine metabolism represented by one AMG, sulfur carrier protein ThiS adenylyltransferase. Besides this pathway, they had a larger proportion of AMGs involved in lysine degradation, peptidoglycan biosynthesis, carbon fixation in photosynthetic organisms, and glutathione metabolism.

When diving into specific AMGs found among the large phages, photosystem II P680 reaction center D1 and D2 proteins were found, which protect photosystem II against photoinhibition and UV-B effects (58, 59). The CpeT protein, a component of phycoerythrin in cyanobacteria, was also found. Phosphoribulokinase was also found in these large phages, which plays a vital role in photosynthetic enzyme activity modulation (60).

### Phage host predictions

The species classification of 329 (16.7%) phages provided some insight into the hosts of the viruses, but most prokaryotic viruses could not be classified. Using a variety of bioinformatic approaches, however, hosts could be predicted for 312 (15.9%) phages. The families of the dominant hosts of phages from both Saint Martin and Cox’s Bazer were Rhodobacteraceae, Flavobacteriaceae, Enterobacteriaceae, and Vibrionaceae (Fig. S2). Saint Martin, however, had a much higher proportion of *Vibrio* host predictions than Cox’s Bazar, as well as the less dominant predicted host families of Alteromonadaceae and Chitinophagaceae. Interestingly Cox’s Bazar phages were predicted to infect hosts of the Rickettsiales order, which is a group commonly associated with disease and parasitism in marine organisms. Cox’s Bazar also contains a number of predicted hosts of the Mycobacteriacea family, members of which are known human pathogens involved in diseases like tuberculosis and leprosy, suggestive of wastewater input.

### NCLDV diversity and function

**Fig 6:**
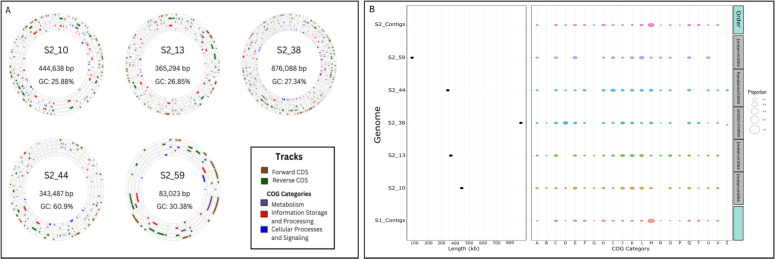
NCLDV genomes and functional potential. (**A**) Genome plots of the five recovered NCLDV genomes, showing GC percentage and total length. Tracks are also shown for forward and reverse coding sequences (CDS) as well as the three major groupings of cluster of orthologous group (COG) categories (clusters of orthologous genes): metabolic, information, and cell processing. (**B**) Functional analysis of the genomes was done by annotating the genomes and unbinned contigs against the GVOG, Pfam, and EggNOG databases using HMMER. What is shown are COG categories and the proportion of genes encoded by each genome or unbinned NCLDV contig in that respective category. COG categories are A, RNA processing; B, chromatin dynamics; C, energy production; D, cell cycle control; E, amino acid metabolism; F, nucleotide metabolism; G, carbohydrate metabolism; H, coenzyme metabolism; I, lipid metabolism; J, translation; K, transcription; L, replication and repair; M, cell membrane biogenesis; N, cell motility; O, post-translational modification; P, inorganic ion transport; Q, secondary structure; T, signal transduction; U, intracellular trafficking; Y, nuclear structure; Z, cytoskeleton.

In total, five NCLDV MAGs were assembled from Cox’s Bazar but none from Saint Martin, although NCLDV contigs were present in Saint Martin as well. Their genome sizes ranged from 83 to 876 kbp. Four belonged to the order Imitervirales and one belonged to Pandoravirales. GC percentages for these genomes ranged from 25.88 to 60.9. Maps of the genomes also show a wide number of coding regions with metabolic functions as well as genes involved in DNA processing (Fig. 6A). Gene counts from the genomes range from 72 to 831, and tRNA counts range from 0 to 10 per genome (Table S2).

Apart from the five NCLDV MAGs we discovered, we also focused on the phylogenetic diversity of the MCP that we could detect in NCLDV contigs to get a broader view of NCLDV diversity in this system (Fig. 5B). A total of 53 MCPs were recovered from our assembled contigs, of which 51 came from Cox’s Bazar and only 2 from Saint Martin, which we compared with reference MCP sequences of cultured NCLDV representatives. The majority of these MCP fell into the Algavirales or Imitervirales order, with one clustering inside the Pandoravirales order. Both MCPs recovered from Saint Martin were found within the Imitervirales order. It is to be noted that multiple copies of MCP are usually present within many NCLDV genomes, and some cases of transfer of this gene across the orders possibly happened, as suggested by the placement of reference genome NCLDVs in our phylogeny. Nevertheless, this gene is frequently used for phylogenetic assessment, given its near-universal presence across a broad range of NCLDVs (61–63). In addition to the 5 MAGs, 39 unbinned NCLDV contigs from Saint Martin and 60 from Cox’s Bazar were recovered and used for functional profiling.

NCLDV genomes from the BoB encode a wide range of functional capabilities, with two of the MAGs and five unbinned NCLDV contigs from both sites having genes involved in carbohydrate metabolism and transport (Fig. 6B). The largest proportion of genes in both sets of unbinned contigs included those involved in cell membrane biogenesis. Two of the giant virus MAGs had proteins for cytoskeleton manipulation which have been described in previous studies (64). Three contained proteins for chromatin modification. Although less common, two of the NCLDV MAGs and two unbinned contigs encoded genes for ion transport.

## DISCUSSION

Marine viruses are ubiquitous in the world’s oceans, and their diversity and abundance typically reflect that of their hosts, whether prokaryotic or eukaryotic (65). As viruses can only reproduce by infecting their hosts, they are tightly linked to processes impacting their hosts’ availability. In a sister study to this one, the microbial communities at each of these sites were characterized (66), which found that Cox’s Bazar had a higher alpha diversity and species richness in terms of both eukaryotic and prokaryotic communities compared to Saint Martin. Likewise, we found viral diversity was higher at Cox’s Bazar compared to Saint Martin, which suggests that a more diverse host pool could result in a more diverse viral pool. However, studies have shown conflicting results in marine environments and also suggest taxonomic resolution plays a role in this relationship (16, 67). Nevertheless, multiple factors could contribute to an observed higher viral diversity, as well as abundance, at Cox’s Bazar. Cox’s Bazar had on average higher temperatures, lower salinity, and higher total dissolved solids, which may play a role in viral diversification, although only salinity was significantly different from Saint Martin. Furthermore, Cox’s Bazar’s proximity to a developed and populated coastline could also enhance viral abundance and diversity here—as multiple sources like freshwater inputs from the rivers and wastewater that flow into the bay could introduce viruses in this environment compared to Saint Martin.

### Differences in viral community

Surprisingly, only 61 phage genomes (3.01%) were shared between two sites only ~60 miles away from each other. Despite being planktonic and movement mediated by oceanic currents, it seems that viruses may exhibit high endemicity in this region, a phenomenon that has previously been reported for phages in the ocean’s surface waters (67, 68). Because of the fairly similar water temperatures and time of sampling, which are known to impact viral community structure (67, 69), other factors could be contributing to the distinctions between the two sites. Furthermore, metagenomes are a snapshot of a community in time, and only two samples were collected at these sites; more would be needed statistically to confirm this. Future studies will be needed that include spatial and temporal sampling schemes, for a comprehensive assessment of the biotic and abiotic drivers of viral community differences in these sites.

One convincing possibility for the striking differences in the viral communities of these sites is that human land usage and water inputs are impacting these sites to different degrees. A study by French et al. (70 ) found that in a river environment, urban development and farming had significant impacts on the virus community present in the water. In our study, the influence of land usage and terrestrial water input may explain the detection of phages associated with prokaryotic hosts that cause plant diseases such as the *Ralstonia* phage, which was identified in high abundance at Cox’s Bazar (71). Furthermore, anthropogenic water inputs to a marine environment typically add nutrients such as phosphorus and nitrogen, which are often thought of as limiting nutrients for photosynthetic organisms in the photic zone (72). These bloom-forming conditions may have resulted in the observed high abundance of cyanophages and *Celluphaga* phages in Cox’s Bazar site compared to Saint Martin. Cyanophages infect cyanobacteria and the abundance of these phages may reflect an increase in cyanobacterial growth from the nutrient input. Additionally, phages infecting the bacterial genus *Celluphaga* could be indicative of large inputs of organic matter; *Celluphaga* spp. mainly consume organic matter from decaying microbial cells and other organic molecules (73, 74). This genus has also been detected as enriched at Cox’s Bazar relative to Saint Martin in the sister study by Akter et al. (66). Although the high abundance of these phages does not always indicate the high abundance of their host, it suggests they are active in these environments, considering the often-quick decay rates of phage particles (75, 76).

### Differences in AMGs and metabolic pathways

The larger viral abundance and diversity at Cox’s Bazar likely reflect the larger diversity and abundance of AMGs found within the genomes of these viruses. AMGs have been shown to be important factors in modulating host metabolism (17). The presence of a broader range of these AMGs at Cox’s Bazar could imply a broader range of metabolisms used by hosts from which these genes were likely acquired - as AMGs have been shown to vary by host diversity (16). Many AMGs are possibly used by viruses to increase virus production by modulating rate-limiting processes in the host (77).

The pathway that was the most differentially abundant in Cox’s Bazar compared to Saint Martin was glutathione metabolism, having 5× more genes found in Cox’s Bazar. This pathway has been shown to be implicated in antioxidant defense, nutrient metabolism, and regulation of cellular events (78), suggesting phages in Cox’s Bazar may have the ability to manipulate these processes in their hosts. Peptidoglycan biosynthesis is another pathway that is only found in Cox’s Bazar. This pathway has been shown to be utilized by some phages during infection as disrupting peptidoglycan cell walls of bacteria could make viral entry easier (79). The presence of this pathway at only one site could suggest, as it has been hypothesized, that there are phage-specific strategies for infection and metabolic reprogramming of host cells (80). It could also be the case that this was a rare pathway and was unable to be detected at Saint Martin due to methodological limitations.

### Large phages

Jumbo phages have gained recent interest for their large sizes and therapeutic potential, being seen as natural antibacterials against pathogenic coral-infecting bacteria (81) as well as their potential to fight human pathogens (82). These phages have previously been characterized based on their genome size, biogeography, and infection strategies (20). Here, we show that there are jumbo phages from multiple distinct phylogenetic groups in the *terL* gene phylogeny of phages, with a large clade particularly enriched in these phages. A similar phylogenetic approach can be used to identify and classify novel jumbo phages as well as looking into the evolutionary history of these large phages in other marine environments. This phylogenetic relationship helped to confirm the presence of jumbo phages at Cox’s Bazar through phylogenetic similarity to known jumbo phages. Jumbo phages are a part of the larger and more diverse viral community at Cox’s Bazar, potentially due to an increased amount of nutrients to support larger host populations. It is important to iterate that recovery of a large number of jumbo phage-specific phylogenetic markers in our study is consistent with the observations that large viruses are typically enriched within the cellular size fraction. Thus, our study re-affirms the importance of investigating the cellular size fraction for the assessment of viral diversity along with the <0.22-μm fraction that is routinely used (83).

Large phages in our study contained a large number of AMGs relative to the small number of identified genomes of 16. Due to their large genome sizes, these phages have a larger capacity to encode AMGs and confer metabolic changes to their host. Among large phage-encoded metabolic potentials from the genomes in this study, we found a high number of AMGs involved in the control of photosynthesis such as photosystem II P680 reaction center D1 and D2 proteins. These genes have been shown to be functional in the host and have been hypothesized to increase phage fitness through supplementing and protecting the host’s photosynthetic machinery (77, 84). Jumbo phages remain a poorly studied group of phages, and further investigations into their full ecological potential are required.

### Viral host prediction

Viral host prediction can be a challenging task when culture-confirmed interactions are not available from the study environment. Our host prediction approach leveraging multiple bioinformatic evidence revealed a large proportion of host-virus interactions within the Rhodobacteraceae. This result is consistent with the fact that Rhodobacteraceae is highly abundant in coastal waters across the world, playing a role in organic matter degradation (85). Interestingly, Cox’s Bazar had host predictions to members of the Rickettsiales order, which is commonly associated with disease and parasitism in marine organisms. Cox’s Bazar also contains a number of hosts predicted from the Mycobacteriaceae family, which is known for causing diseases such as tuberculosis and leprosy in humans (86). These pathogenic hosts are likely the result of the many anthropogenic influences in the Cox’s Bazar waterways. Microbial analysis of Cox’s Bazar also suggested that these waters could be harboring pathogenic organisms due to the significant number of identified virulence genes (66).

### NCLDV abundance and diversity

While only Cox’s Bazar samples had a high enough abundance of NCLDVs to recover MAGs, NCLDV contigs were still found in Saint Martin, and we cannot rule out the possibility that biases in assembly or sequencing may have hidden more NCLDV MAGs from Saint Martin. The majority of *mcp*s found in the data are from the orders Algavirales and Imitervirales, which are the two largest orders of NCLDVs and are generally the most abundant in coastal waters (87). The large genomes of NCLDVs have been shown to greatly alter host metabolism as they contain many genes that mimic host homologs and can hijack host machinery to turn the host’s metabolism towardmaking viruses (24). Many of these genes are unique to NCLDVs in the virus community and are likely acquired from cellular hosts (88). Functional annotation of our NCLDV genomes revealed genes such as those involved in DNA packaging (histones), cytoskeletal manipulation, and ion transport. It is hypothesized that NCLDV histones mimic host histones to protect viral DNA from degradation, forming similar structures to nucleosomes (89). Ion transporters have long been found in viruses and likely play a role in viral entry through depolarization of the membrane, and nutrient acquisition to provide the host with additional nutrients to support viral replication (90). Cytoskeletal manipulation proteins have been suggested to be used by NCLDVs to hijack the host’s vesicular trafficking, bringing cargo to the viral factory for viral assembly and production (91, 92). Our data on the NCLDVs represent the first report of NCLDVs in the Bay of Bengal, highlighting their potential role in the microbial food web in this region and adds to the growing body of knowledge on the diversity and genomic potential of NCLDVs in the world’s ocean.

### Limitations

Despite the valuable insights gained from this study, it is important to acknowledge and address several limitations that may have influenced the findings. The limited number of sites by which to compare viral populations limits the scope of this study, as only two sites were selected in this large bay. The pooling of the two samples from these two sites also limits our ability to evaluate drivers of the viral community at a finer spatial scale. Additionally, more physicochemical parameters would have been helpful in order to provide further evidence for anthropogenic influences and support hypotheses regarding the differences in the viral communities. However, the primary motivation for this study was to evaluate the diversity of viruses and their hosts in this understudied, yet highly important ecosystem. Future analyses with more samples collected at a higher spatial resolution will be necessary to discover the drivers of virus community structure and dynamics. The current repertoire to classify and identify viral hosts is also limited, and we could classify and identify host affiliation of only a small proportion of the viruses. It is also likely that many of the viruses found in this fraction are not necessarily associated with a host, as there is a growing concept of viral grazing and ingestion by protists (93). In spite of these limitations, this study provides the necessary foundation for future research on the microbial and viral communities in the BoB.

### Conclusion

Our study aimed to address the knowledge gap regarding the viral community diversity and structure within an understudied ecosystem, the BoB. Given the region’s susceptibility to frequent monsoons, flooding, and vulnerability to sea-level rise, the BoB holds research significance as its neighboring nations grapple with these challenges. Considering the crucial role of microbial populations in supporting fisheries that sustain numerous livelihoods, our research on a portion of the BoB’s virus population contributes to the broader understanding of the factors that constrain microbial population dynamics and their contribution to the food web in this ecosystem. Our study underscores the importance of continued research in comprehending the intricate dynamics and ecological significance of microbial populations in sustaining the blue economy, as well as the anthropogenic influences affecting viral and microbial dynamics and abundance in this region. In addition, the detection of giant viruses and large phages in the Bay of Bengal underscores the need to extend marine virological research into less-studied ecosystems, which will enhance our comprehension of the rich diversity of marine viruses and better elucidate their evolutionary histories. Finally, this work will be a crucial reference for future research on deciphering the viral impact on microbial food web dynamics in the Bay of Bengal.

## Data Availability

Raw sequence reads are available at the National Center for Biotechnology Information database under the bioproject number PRJNA936489. Genomes of NCLDVs and large phages, as well as the information used to make phylogenetic trees, functional annotations, and code used to make the plots, can be obtained from https://figshare.com/projects/Phylogenetic_diversity_and_functional_potential_of_large_and_cell-associated_viruses_in_the_Bay_of_Bengal/171777. Bootstrapped versions of maximum likelihood *terL* and *mcp* trees in Newick format are also provided in the same repository. The pipeline for identification of giant virus genomes can be found at https://github.com/BenMinch/PIGv.

## SUPPLEMENTAL MATERIAL

Supplemental Material msphere.00407-23-s0001.pdf Supplemental figures and table.

ASM does not own the copyrights to Supplemental Material that may be linked to, or accessed through, an article. The authors have granted ASM a non-exclusive, world-wide license to publish the Supplemental Material files. Please contact the corresponding author directly for reuse.
