# Supplementary material for: Phylogenetic diversity and functional potential of large and cell-associated viruses in the Bay of Bengal
Source: mSphere. 2023 Oct 30;8(6):e00407-23. doi: 10.1128/msphere.00407-23 (PMC10732071; doi:10.1128/msphere.00407-23)
Supplement: Supplemental Material — Supplemental figures and table. [file msphere.00407-23-s0001.pdf]

Supplementary data for

Phylogenetic diversity and functional potential of large and cell-associated viruses in the Bay of Bengal

This file includes:

Legends for Supplementary Figure 1, 2, and Table S1

Figure S1. Large phage metabolic potential. A barplot showing auxiliary metabolic genes found in the binned and unbinned large phage genomes using VIBRANT. Colors represent broader metabolic pathways.

Figure S2. Prokaryotic Virus-Host Prediction. Host prediction was performed using iPHOP and represented here is the predicted number of virus-host pairs to the classification level of host family. In total 312 Host-Virus pairs were predicted using this method which represents about 16% of our total viral populations identified.

Table S1. Sequencing and Assembly Statistics.

Table S2. NCLDV Genome Overview. Information about recovered genomes including size, classification, GC percentage, gene count, coding percentage, NCLDV marker genes, and tRNAs.

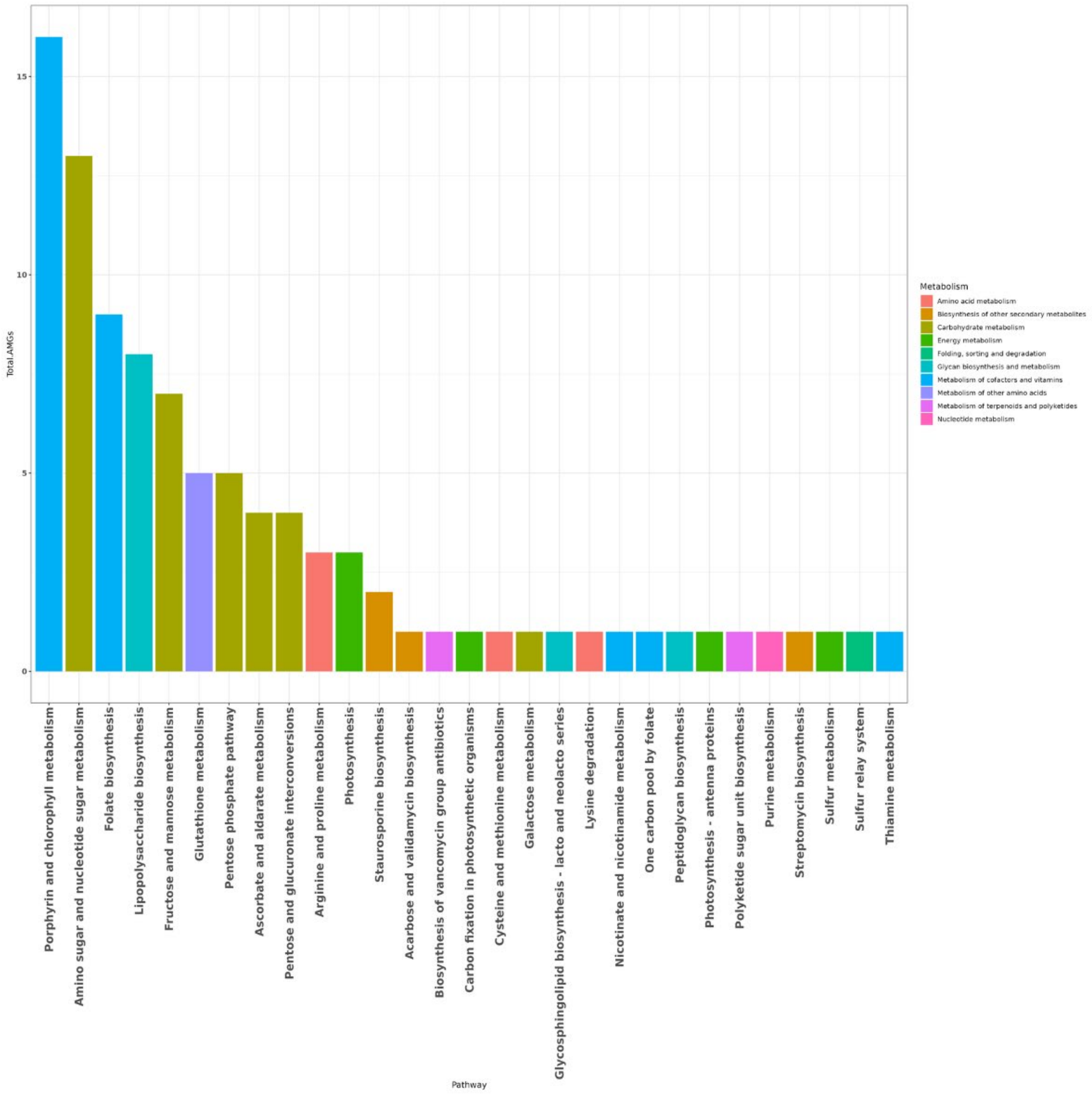

Figure S1. Large phage metabolic potential. A barplot showing auxiliary metabolic genes found in the binned and unbinned large phage genomes using VIBRANT. Colors represent broader metabolic pathways.

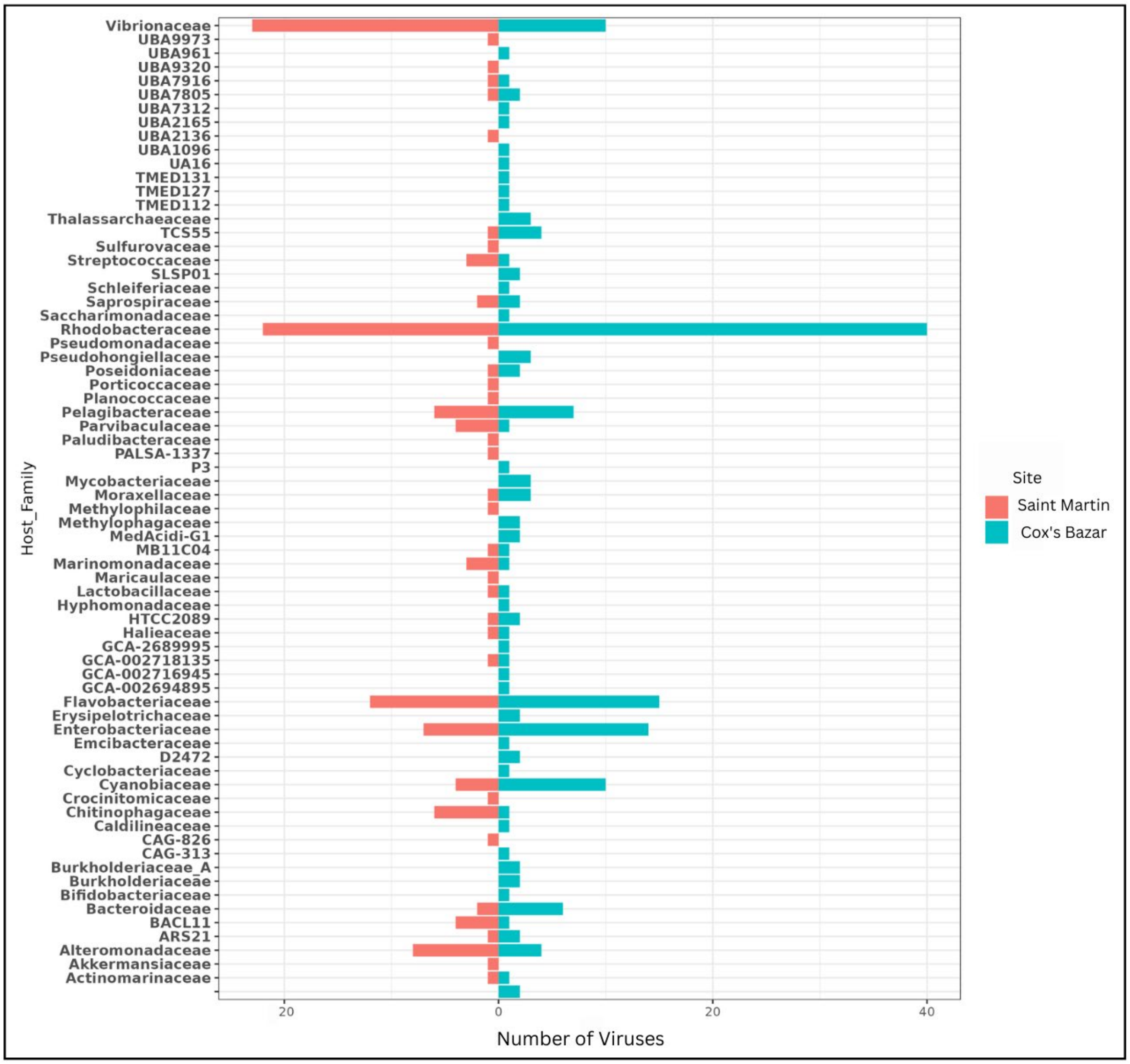

Figure S2. Prokaryotic Virus-Host Prediction. Host prediction was performed using iPHOP and represented here is the predicted number of virus-host pairs to the classification level of host family. In total 312 Host-Virus pairs were predicted using this method which represents about 16% of our total viral populations identified.

Assembly

Sample	Number of Contigs	Average Length	Total Length
Saint Martin	201998	1338.4	270355519
Cox's Bazar	187899	1268.6	238377094

Paired-end reads

Sample	Raw Reads	Trimmed Reads	Percent kept
Saint Martin	36.8 million	33.94 million	92.20%
Cox's Bazar	34.4 million	31.4 million	92.37%

Table S1. Sequencing and Assembly Statistics.

Genome	Size (bp)	GC %	Gene Count	Coding %	tRNAs	NCLDV Marker Genes	Family	Order
S2_10	444638	25.88	290	93.53	10	9	Mesomimiviridae	Imitervirales
S2_59	83023	30.38	72	90.83	1	4	Mesomimiviridae	Imitervirales
S2_13	365294	26.85	313	91.52	9	8	Mesomimiviridae	Imitervirales
S2_38	876088	27.34	831	92.85	0	8	Mimiviridae	Imitervirales
S2_44	343487	60.9	294	87.94	0	4	incertae_sedis	Pandoravirales

Table S2. NCLDV Genome Overview. Information about recovered genomes including size, classification, GC percentage, gene count, coding percentage, NCLDV marker genes, and tRNAs.
